# Initial pen and field assessment of baits to use in oral rabies vaccination of Formosan ferret-badgers in response to the re-emergence of rabies in Taiwan

**DOI:** 10.1371/journal.pone.0189998

**Published:** 2018-01-02

**Authors:** Ryan M. Wallace, Yuching Lai, Jeffrey B. Doty, Chen-Chih Chen, Neil M. Vora, Jesse D. Blanton, Susan S. Chang, Julie M. Cleaton, Kurtis J. C. Pei

**Affiliations:** 1 United States Centers for Disease Control and Prevention. Atlanta, GA, United States of America; 2 Epidemic Intelligence Service, Centers for Disease Control and Prevention (CDC), Atlanta, United States of America; 3 Department of Environmental and Hazards-Resistant Design, Huafan University, Shiding, New Taipei, Taiwan; 4 Institute of Wildlife Conservation, National Pingtung University of Science & Technology, Neipu, Pingtung, Taiwan; 5 Bureau of Animal and Plant Health Inspection and Quarantine, Council of Agriculture. Taipei, Taiwan; 6 Oak Ridge Institute for Science and Education. Oak Ridge, TN, United States of America; Wistar Institute, UNITED STATES

## Abstract

**Background:**

Taiwan had been considered rabies free since 1961, until a newly established wildlife disease surveillance program identified rabies virus transmission within the Formosan ferret-badger (*Melogale moschata subaurantiaca*) in 2013. Ferret-badgers occur throughout southern China and Southeast Asia, but their ecological niche is not well described.

**Methodology/Principle findings:**

As an initial feasibility assessment for potential rabies control measures, field camera trapping and pen assessment of 6 oral rabies vaccine (ORV) baits were conducted in Taiwan in 2013. 46 camera nights were recorded; 6 Formosan ferret-badgers and 14 non-target mammals were sighted. No baits were consumed by ferret-badgers and 8 were consumed by non-target mammals. Penned ferret-badgers ingested 5 of the 18 offered baits. When pen and field trials were combined, and analyzed for palatability, ferret-badgers consumed 1 of 9 marshmallow baits (11.1%), 1 of 21 fishmeal baits (4.8%), 0 of 3 liver baits, and 3 of 3 fruit-flavored baits. It took an average of 261 minutes before ferret-badgers made oral contact with the non-fruit flavored baits, and 34 minutes for first contact with the fruit-based bait. Overall, ferret-badgers sought out the fruit baits 8 times faster, spent a greater proportion of time eating fruit baits, and were 7.5 times more likely to have ruptured the vaccine container of the fruit-based bait.

**Conclusions/Significance:**

Ferret-badgers are now recognized as rabies reservoir species in China and Taiwan, through two independent ‘dog to ferret-badger’ host-shift events. Species of ferret-badgers can be found throughout Indochina, where they may be an unrecognized rabies reservoir. Findings from this initial study underscore the need for further captive and field investigations of fruit-based attractants or baits developed for small meso-carnivores. Non-target mammals’ competition for baits, ants, bait design, and dense tropical landscape represent potential challenges to effective ORV programs that will need to be considered in future studies.

## Introduction

Enzootic circulation of rabies virus in Taiwanese dogs was recognized as early as 1903 [1]. Through mass vaccination and other control efforts this canine variant was eliminated from Taiwan in 1961 [2]. For the following 51 years the island was considered rabies-free and compulsory dog vaccination was deregulated. In 2012, a wildlife disease detection program was established at several wildlife rescue centers. Within one year of implementation, enzootic rabies virus transmission was recognized in Formosan ferret-badgers (*Melogale moschata subaurantiaca*) [1]. Phylogenetic analysis of the Formosan ferret-badger rabies virus variant indicated that this variant shifted from Taiwanese dogs to ferret-badgers prior to 1950, and went unrecognized in the ferret-badger population for over 50 years [2].

At least two independent ‘dog to ferret-badger’ host-shift events are known to have occurred (China and Taiwan) [2–4]. Ferret-badger species are widely distributed throughout Indochina, where canine rabies is also highly enzootic [5]. To-date, robust disease surveillance in ferret-badgers has only been conducted in China and Taiwan, with enzootic rabies transmission cycles detected in both countries. Therefore, recognition of ferret-badger rabies reservoirs and efforts to control spread have potentially far-reaching implications.

Oral rabies vaccination (ORV) has been proven a successful method for wildlife rabies control [6]. These programs require an interdisciplinary approach combining ecology, virology, vaccinology, and epidemiology, as well as other disciplines. Considering that successful elimination of rabies has been achieved in other wildlife reservoir populations, field and pen-trials were conducted to determine if any of six pre-selected ORV baits are both attractive and palatable at high rates in ferret-badgers.

## Methods

**Fig 1:**
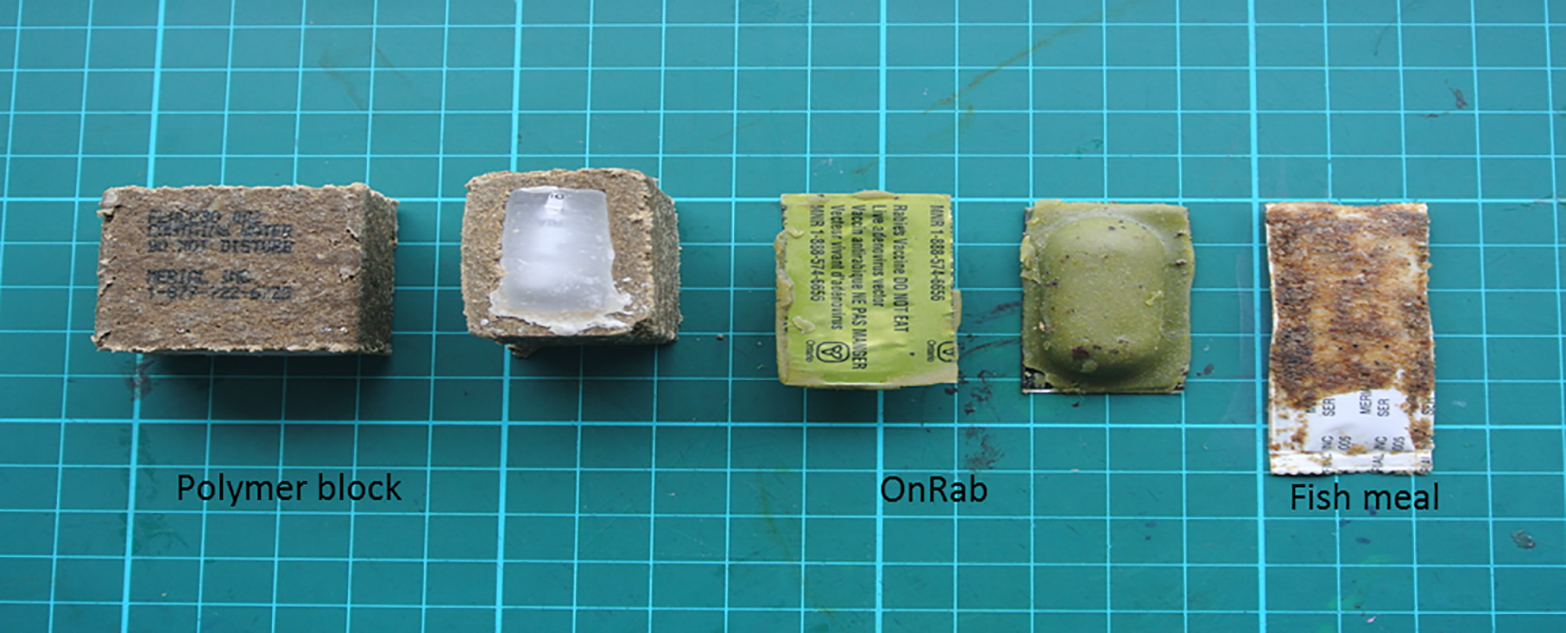
Three oral rabies vaccination bait constructs assessed for feasibility of use in ferret-badgers.

This study was conducted in Taiwan during a six week period in 2013. A pen trial with three captive wild-caught ferret badgers and a field-based camera trap study were employed to evaluate six oral bait constructs containing a placebo (saline) for attractiveness, palatability, and delivery potential (Fig 1).

### Bait formulation

**Ultra-lite (UL)** baits (Artemis Technologies, Guelph, Ontario, Canada) have been used in raccoons (*Procyon lotor*) and skunks (*Mephitis mephitis*). The vaccine container consisted of a polyvinyl chloride blister-pack containing placebo saline coated with a waxy mixture and artificial marshmallow flavoring. The UL bait measured 4.0 cm x 2.2 cm x 1.0 cm and weighed approximately 4.3 grams [7].

**Fishmeal Polymer Block (PB)** baits (Merial, Athens, GA) were designed for use in raccoons and coyotes [8]. The bait measured 3.3 cm x 3.2 cm x 2.2 cm and was comprised of fishmeal based polymer, surrounding a wax block containing placebo saline. The PB bait weighed approximately 24 grams.

**Coated Sachet (CS)** baits (Merial, Athens, GA) were designed for use in raccoons and coyotes [8]. The bait consisted of a polyethylene sachet that contained the placebo saline and was covered in a light adhesive wax and fishmeal crumbles. The CS bait measured 6.4 cm x 1.9 cm x 0.3 cm and weighed approximately 4 grams.

**Rabigen (RG)** baits (Virbac Laboratories, Carros, France) were designed for use in red foxes and raccoon dogs. The RG bait had a vaccine container consisting of a PVC/aluminum sachet containing placebo saline, which was enclosed within a matrix made of mineral fat and fishmeal. The baits measured 4.9 cm x 4.4 cm x 1.5 cm and weighed 28 grams.

**Rabidog (RD)** baits (Virbac Laboratories, Carros, France) were designed for use in dogs. The vaccine container consisted of a PVC/aluminum sachet containing placebo saline, which was enclosed within a chicken liver bait matrix. The RD baits measured 4.4 cm x 1.6 cm and weighed 23 grams.

**IDT-Plum (PL)** bait (IDT Biologika, Dessau Rosslau, Germany) was designed for use in small meso-carnivores. This bait consisted of a plastic sachet that containing placebo saline, and was coated in a jellied texture flavored with plum fruit. The PL bait measured 3 cm × 1.5cm × 1cm and weighed approximately 6 grams. This bait is not currently commercially available. This bait was only used for pen-trials.

### Field trials

**Fig 2:**
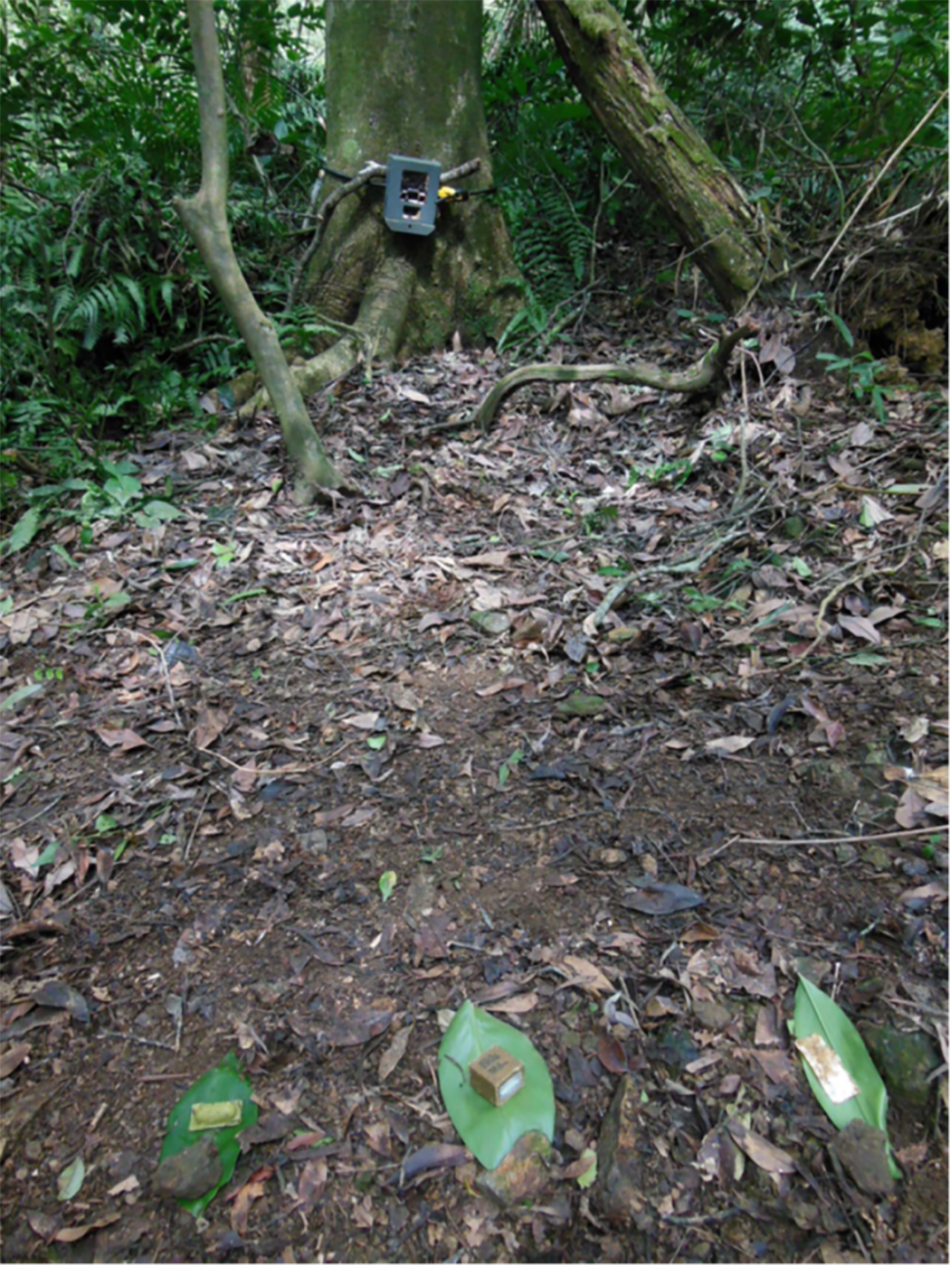
Set-up used for testing the five bait types used in field trials. Cameras were set at dusk and retrieved in the morning. Data collected from images of ferret-badgers and non-target species were assessed on four criteria: 1) Animals were observed but had no interaction with baits; 2) Animals investigated the baits (attractiveness); 3) Animals had direct physical contact with the baits (attractiveness); 4) Animals were observed to have ingested the entire bait or there was evidence that the vaccine container was punctured (palatability).

Field trials were conducted at Luanshan, Shoufeng, Shiding, and Chenggong sites between mid-August and September. UL, PB, and CS bait types were used at the Luanshan, Shoufeng and Shiding sites and were placed 18-inches apart (Fig 2). RG and RD baits constructs were used at the Chenggong site. A total of 8 camera trapping nights were conducted at both the Luanshan and Shoufeng sites and 26 camera nights at Shiding. RG and RD baits were placed several feet apart, within view of field cameras, for a total of four nights.

### Pen trials

Pen trials were conducted at the same time as field trial dates. Baits were offered to three wild-born, captive ferret-badgers, housed in separate but adjoining 1.5m wide, 3.3m long, 3m high pens. One of each UL, PB, RG, RD, PL, and CS bait types were offered. Video cameras captured the full pen width and the first 1.7m of its length. The baits were placed in the pens at dusk (around 1700 hr) and those remaining were removed at 0800 hr.

Bait exposure time was defined as the period (seconds) in which a bait could be seen, and was censored if the bait was removed from camera view. “Attractiveness” was measured as the amount of time the ferret-badger was sniffing or otherwise investigating the baits, including physical contact. “Palatability” was defined as the amount of time a bait was in contact with oral mucosa, including chewing or carrying a bait.

### Ethics statement

All animal activities were conducted under the US CDC IACUC protocol number 2498FRAMULX. We followed guidelines from the American Society of Mammalogists for the use of wild mammals in Research [9].

## Findings from field trials

Animals were recorded on 19 out of a total of 46 camera nights at the four field sites. Six ferret-badgers, 3 crab eating mongoose (*Herpestes urva formosanus*), 2 Reeves’ muntjac (*Muntjacus reevesi micrurus*), 1 masked palm civet, 1 small Chinese civet (*Viverricula indica pallida*), 2 domestic dogs (*Canis lupus familiaris*), 2 domestic cats (*Felis catus*), 2 rats (*Niviventer coxingi*), and 1 squirrel (*Callosciurus erythraeus*) were observed (Table 1). No ferret-badgers were observed within camera view of the RG and RD baits.

**Table 1:** Comparison of five oral rabies vaccines in four field sites, Taiwan 2013. Information was collected on whether the baits were investigated, contacted, ingested or removed, and ruptured.

Camera Site ^a^	Terrestrial mammals observed		Baits Offered	Baits investigated	Baits contacted	Baits ingested or removed	Vaccine Container Ruptured
Luanshan	Dog	*Canis lupus familiaris*	UL, CS, PB	UL, CS, PB	UL, CS, PB	CS, PB	CS, PB
Luanshan	Dog	*Canis lupus familiaris*	UL, CS, PB	UL, CS, PB	UL, CS, PB	UL, CS, PB	UL, CS, PB
Shoufeng	Cat	*Felis catus*	UL, CS, PB	UL, CS, PB	CS, PB	none	none
Shoufeng	Cat	*Felis catus*	UL, CS, PB	UL, CS, PB	CS, PB	PB	PB ^b^
Shoufeng	Civet	*Paguma larvata*	UL, CS, PB	CS, PB	PB	none	none
**Shiding**	**Ferret-badger**	***M*. *moschata subaurantiaca***	UL, CS, PB	**none**	**none**	**none**	**none**
**Shiding**	**Ferret-badger**	***M*. *moschata subaurantiaca***	UL, CS, PB	**none**	**none**	**none**	**none**
**Shiding**	**Ferret-badger**	***M*. *moschata subaurantiaca***	UL, CS, PB	**none**	**none**	**none**	**none**
**Shiding**	**Ferret-badger**	***M*. *moschata subaurantiaca***	UL, CS, PB	**none**	**none**	**none**	**none**
**Shiding**	**Ferret-badger**	***M*. *moschata subaurantiaca***	UL, CS, PB	**none**	**none**	**none**	**none**
**Shiding**	**Ferret-badger**	***M*. *moschata subaurantiaca***	UL, CS, PB	**CS, PB**	**PB**	**none**	**none**
Shiding	Small Chinese civet	Viverricula indica pallida	UL, CS, PB	none	none	none	none
Shiding	Tree squirrel	*Callosciurus erythraeus*	UL, CS, PB	none	none	none	none
Shiding	Rat	*Niviventer coxingi*	UL, CS, PB	none	none	none	none
Shiding	Rat	*Niviventer coxingi*	UL, CS, PB	none	none	none	none
Chenggong	Crab-eating moongoose	*Herpestes urva*	RG, RD	RG	none	none	none
Chenggong	Crab-eating moongoose	*Herpestes urva*	RG, RD	RG	RG	RG	RG ^b^
Chenggong	Crab-eating moongoose	*Herpestes urva*	RG, RD	RD	RD	RD	RD
Chenggong	Muntjac	*Muntiacus reevesi*	RG, RD	RG	none	none	none
Chenggong	Muntjac	*Muntiacus reevesi*	RG, RD	RD	none	None	none

^a^ = A total of 46 camera nights was recorded during the study period.

^b^ = Bait was not found and was presumed to have been ingested.

(UL–UltraLight) (CS–Coated Sachet) (PB–Polymer Block) (RG–Rabigen) (RD–Rabidog)

**Fig 3:**
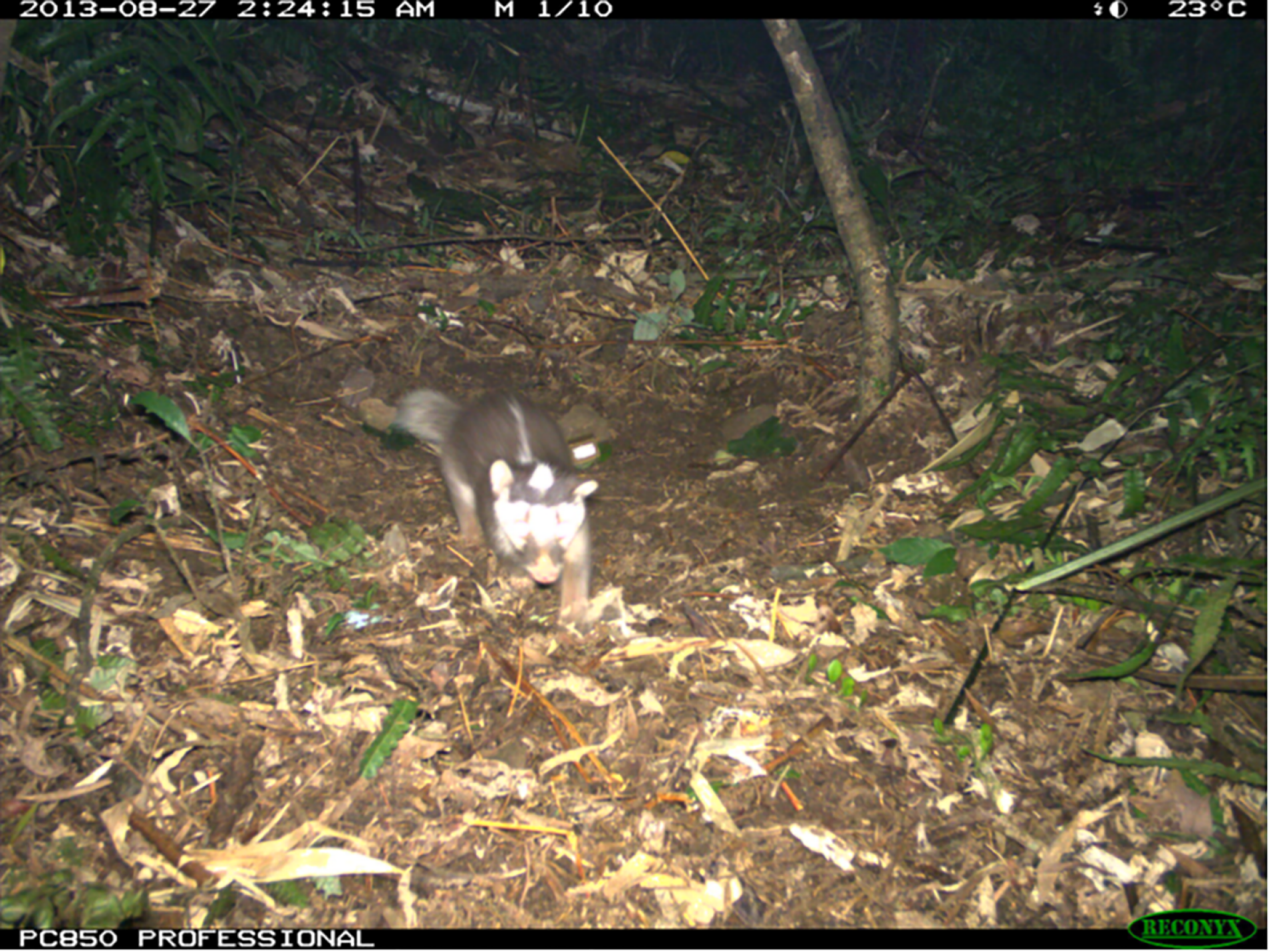
Ferret-badger photographed at field camera site.

The camera capture rate for the target species was 13.0% (6 of 46) (Fig 3). Ferret-badgers made physical contact with 1 of the 18 baits they encountered and no ferret-badgers ingested or punctured the vaccine containers. Fourteen non-target mammals made contact with 14 of 32 baits they encountered (43.8%) and 8 were ingested (25.0%). Ten of 134 baits (7.5%) placed in the field were covered by ants 12 hours after placement.

## Findings from pen trials

A total of 72 hours of video was recorded in assessing the 6 bait types from 3 ferret-badgers, for a total of 18 bait offerings. Ferret-badgers made oral contact with all 3 PL baits (100%), 7 of the 9 fishmeal-based baits (PB, CS, RG) (77.8%), 2 of 3 marshmallow baits (UL) (66.6%), and 2 of 3 liver baits (RD) (66.6%) (Table 2). All 3 PL baits (100%) were fully ingested, compared to only 33.3% of marshmallow, 11.1% of fishmeal, and 0% of liver based baits.

**Table 2:** Comparison of 6 oral rabies vaccination baits in a pen trial, Taiwan 2013.

Bait Type	Pen (Exposure Time) ^a^	Attractiveness: ^b^ Seconds (%)	Contact Events	Palatability: ^c^ Seconds (%)	Oral Contact with Bait	Time to First Oral Contact: ^d^ Seconds	Blister Pack Ruptured
Ultra Lite (Marshmallow)	FB A (43,200)	34 (0.1%)	4	13 (38.2%)	**Yes**	4,319	**Yes**
	FB B (43,200)	0 (0.0%)	0	0 (0.0%)	No	43,200	No
	FB C (33,236)	119 (0.4%)	8	57 (47.92%)	**Yes**	3,656	No
	***Average***	***51 (0*.*1%)***	***4***	***23 (45*.*1%)***	***2 of 3***	***17*,*058***	***1 of 3***
Polymer Block (Fishmeal)	FB A (638)	10 (1.6%)	2	3 (30.0%)	**Yes**	636	**Yes** ^**e**^
	FB B (43,200)	0 (0.0%)	0	0 (0.0%)	No	43,200	No
	FB C (24,102)	44 (0.2%)	6	42 (95.5%)	**Yes**	11,328	No
	***Average***	***18 (0*.*1%)***	***3***	***15 (83*.*3%)***	***2 of 3***	***18*,*388***	***1 of 3***
Coated Sachet (Fishmeal)	FB A (2,316)	18 (0.8%)	2	5 (27.82%)	**Yes**	2,316	No
	FB B (43,200)	0 (0.0%)	0	0 (0.0%)	No	43,200	No
	FB C (43,200)	58 (0.1%)	5	35 (60.3%)	**Yes**	3,039	No
	***Average***	***25 (0*.*1%)***	***2***	***13 (52*.*0%)***	***2 of 3***	***16*,*185***	***0 of 3***
Rabigen (Fishmeal)	FB A (32,350)	89 (0.3%)	1	4 (4.5%)	**Yes**	32,346	No
	FB B (8,045)	459 (5.7%)	3	450 (98.0%)	**Yes**	6,323	No
	FB C (44,630)	26 (0.1%)	3	26 (100%)	**Yes**	3,026	No
	***Average***	***191 (0*.*7%)***	***2***	***160 (83*.*8%)***	***3 of 3***	***13*,*898***	***0 of 3***
Rabidog (Liver)	FB A (27,400)	5 (0.0%)	0	0 (0.0%)	No	27,400	No
	FB B (9,562)	27 (0.3%)	2	8 (29.6%)	**Yes**	9,369	No
	FB C (26,201)	150 (0.6%)	6	150 (100%)	**Yes**	1,590	No
	***Average***	***61 (0*.*3%)***	***3***	***53 (86*.*9%)***	***2 of 3***	***12*,*786***	***0 of 3***
IDT-Plum (Fruit)	FB A (3,513)	78 (2.2%)	2	78 (100%)	**Yes**	2,820	**Yes**
	FB B (3,480)	175 (5.0%)	2	171 (97.7%)	**Yes**	3,309	**Yes**
	FB C (909)	48 (5.3%)	3	48 (100%)	**Yes**	138	**Yes**
	***Average***	***100 (3*.*8%)***	***2***	***99 (99*.*0%)***	***3 of 3***	***2*,*089***	***3 of 3***

*a*–Exposure time is defined as the amount of time in seconds the baits were within camera view, within the pens.

*b*–Attractiveness is defined as the amount of time the animals were investigating or in physical contact with bait.

*c*–Palatability is the amount of time the animal spent either chewing or carrying the bait by mouth.

*d–*Time to first oral contact was the amount from bait introduction to oral engagement (i.e. chewing or carrying)

*e*–Bait was not located and was presumed to have been ingested

**Fig 4:**
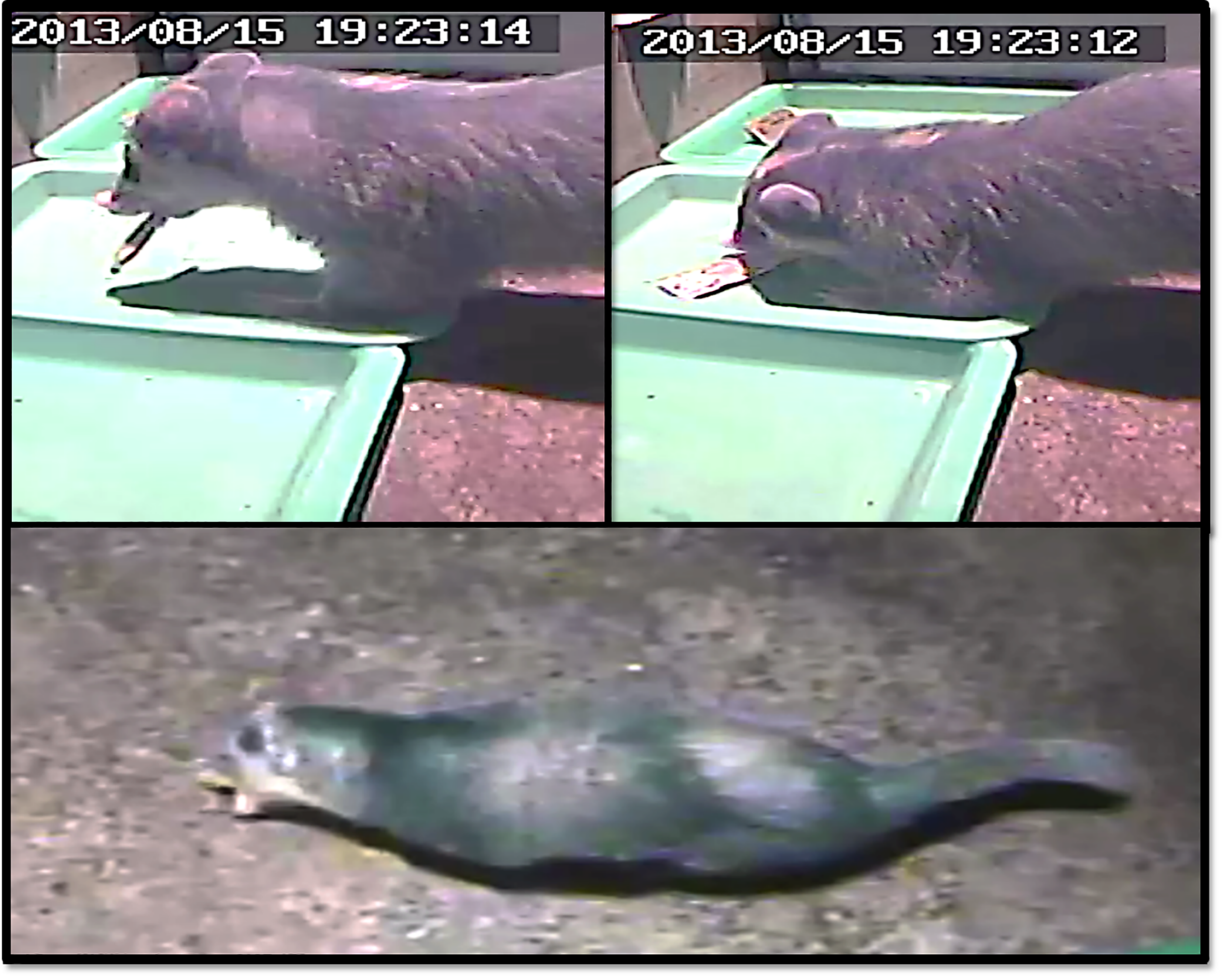
Captive ferret badger feeding stations and consumption mechanics.

Overall, ferret-badgers showed little interest in the UL, PB, CS, RG, and RD baits (range: 0.1–0.7% of exposure time, yet spent 3.8% of their bait exposure time interacting with the PL baits (Rate Ratio: 7.5; Range: 5.4–38.0). The average time to first oral contact with the PL bait was 34 minutes, 6.1–8.8 times faster than other bait constructs. Regardless of bait-type, ferret-badgers were not observed using forepaws to manipulate baits during oral contact events. Oral manipulation and chewing of baits was performed by pinning the bait to the ground with the mouth (Fig 4).

## Discussion

In areas where canine rabies has been controlled, recognition of the disease in wildlife has increased over time, placing people and domestic animals at risk for exposure [3, 10]. In China, where the ferret-badger has been recognized as a rabies reservoir for several decades, this species has been associated with both human and livestock rabies deaths [3]. Ferret-badgers occur in a wide-range of habitats throughout Indochina, which overlap with areas of high enzootic canine rabies activity. It is likely that other ferret-badger host-shift events have occurred (or may occur), but are not currently recognized due to insufficient rabies surveillance capacity in much of the region. This study has identified a potential vaccine bait type for targeting ferret-badgers for rabies control. As these findings are preliminary and based on small sample sizes, further evaluation is required to determine the most effective bait for delivering oral rabies vaccine to be an effective strategy for rabies control in ferret-badgers in Taiwan and perhaps elsewhere in Indo-China.

### ORV: Attractants and palatability

Successful ORV baits must be capable of attracting the target species and must be consumed, while also remaining less appealing to non-target animals and arthropods. No ferret-badgers were captured on cameras at 3 of the 4 field sites. This may be an indication of poor attractiveness for all 5 bait types assessed in the field. Diet preference studies have shown that ferret-badgers in mainland China commonly consume fruits [11], while in northern Taiwan, their primary diet components were insects, earthworms and amphibians [12]. However, regional variation in population densities could also be a contributing factor to this result. After completion of the present field study, a survey at Luanshan and Chenggong showed a more than 90% decline in ferret-badger population following a rabies epizootic in 2013 [13]. Similar population declines have also been reported in other reservoir species, following rabies epizootic events [14]. In comparison, all ferret-badger sightings in the field study occurred at the Shiding site, which was not affected by the rabies epizootic. Despite the low capture rate, six ferret-badgers were seen on-camera, within a meter of the five bait types assessed, yet only one of the five approached the baits. None of the baits were ingested.

Field trials which assessed baits consisting of marshmallow, fishmeal, and liver-based attractants were unsuccessful. Unfortunately the only bait type composed of a fruit-based attractant, the PL bait, was not available for use in the field. The PL bait was assessed only in the pen-trial setting. Despite this disadvantage, the PL bait was preferred by penned ferret badgers compared to the other baits tested. When pen and field trials were combined and analyzed for palatability, ferret-badgers consumed only 2 of 33 non-fruit based baits, compared to consumption of all 3 of the fruit-based PL bait. Additionally, oral engagement occurred in 34 minutes with the fruit-based PL bait, compared to 261 minutes on average for baits with non-fruit attractants. Overall, ferret-badgers sought out the PL baits 8 times faster, spent a greater proportion of time eating the PL baits, and were 7.5 times more likely to have ruptured the PL vaccine container. The finding that fruit-based attractants may be more suitable for ferret-badgers is consistent with their natural diet, which consists of fruits and insects [15]. The size of the PL bait may also contribute to its relative success among ferret badgers; it was the smallest, although the UL and CS baits were lighter.

### ORV: Bait design

ORV baits that have shown great success in attracting target species and palatability have still failed when the bait delivery system was not optimized for the target species [16]. Five of the baits used in this study were designed for use in larger meso-carnivores (dogs, foxes, raccoon dogs). These bait types have not shown as much success in skunks and mongooses, partly due to the food handling and feeding behavior of these species [16]. Skunks and mongooses pin the bait to the ground and often roll baits during consumption, which often results in spillage of the bait contents and limited exposure in the oral cavity. Subjective observations from film of ferret-badger interaction with the baits indicates that their food manipulation and consumption is somewhat similar to skunks and mongooses (Fig 4). Had these larger UL, PB, and CS baits been more successful in terms of palatability, the design of these baits still may have been inadequate for vaccine delivery.

### ORV: Bait competition

Ferret-badgers co-exist with many other native small carnivores in their natural habitats and this study found high levels of competition for the baits among competitor species, particularly dogs, cats and mongooses [13]. The diversity of competing fauna in ferret-badger habitats will need to be considered when developing ORV baits and strategies. Vaccination programs must also consider the potential impacts associated with dispersing vaccine-laden baits on endangered endemic species in Taiwan such as the leopard cat (*Prionailurus bengalensis*) and otter (*Lutra lutra*) [17]. Additional competition may also come from arthropods, which damaged the attractant coating in 7.5% of baits.

## Conclusion

The host shift discovery of rabies from the dog to the ferret-badger in Taiwan may not have been an isolated event. Phylogenetic and observational evidence suggests that other ‘dog to ferret-badger’ host-shift events have occurred, and might occur in the future. Development of effective methods for rabies control in these poorly understood species may have far-reaching public health and wildlife conservation implications for countries where ferret-badgers occur. Findings from this initial study, considering its stated limitations, suggest that baits compatible with the natural diet and consumption mechanics of small meso-carnivores should have a greater chance for success. Continued research is needed on ferret-badger ecology, habitat use, population dynamics, and movements. This information, in tandem with more comprehensive studies to identify optimal baits, attractants, and vaccines is necessary to determine if ferret-badger rabies control is achievable.

## Supporting information

S1 Table Skull morphological comparison of meso-carnivore rabies reservoirs, including the Formosan ferret-badger. (DOCX)
