# Supplementary material for: Initial pen and field assessment of baits to use in oral rabies vaccination of Formosan ferret-badgers in response to the re-emergence of rabies in Taiwan
Source: PLoS One. 2018 Jan 2;13(1):e0189998. doi: 10.1371/journal.pone.0189998 (PMC5749709; doi:10.1371/journal.pone.0189998)
Supplement: S1 Table — (DOCX) [file pone.0189998.s001.docx]

| **Species** | **Scientific name** | **Body weight (kg)** | **Total Skull length (mm)** | **Maximum Skull width (mm)** | **Main diet** | **Reference** |
| --- | --- | --- | --- | --- | --- | --- |
| Coyote | *Canis latrans* | Male: 13  Female: 11.5 | 191.00 | 95.01 | Carnivorous | [14]  [15] |
| Red fox | *Vulpes vulpes* | 3.0 – 11.0 | 146.31 | 74.39 | Carnivorous: Small mammals (voles, rabbits, squirrels, mice) | [14] |
| Raccoon | *Procyon lotor* | Male: 5.4  Female: 4.6 | Male :110.3  Female: 105.5 | Male :71.5  Female: 66.7 | Omnivorous: Rodents, crayfish, fruit, berries, vegetation | [14, 16] |
| Striped skunk | *Mephitis mephitis* | 1.2 - 6.3 | 77.7 | 44 | Omnivorous: Insect, small mammals, fish, crustaceans, fruits, nuts, leaves, grasses, carrion | [17, 18] |
| Small Asian mongoose | *Herpestes javinicus* | 0.5 – 0.8 | Male: 70  Female:68 | Male: 40  Female: 38 | Insects, rodent, birds, reptiles, amphibians | [19] |
| Chinese ferret-badger | *Melogale moschata* | 0.8 - 1.6 | Male: 77  Female:75 | Male: 43.4  Female:43.1 | Omnivorous: Insects, earthworms snails, frogs, carcasses of small birds and mammals, eggs, fruit | [20] |
| Formosan ferret-badger | *Melogale moschata Subaurantiaca* | 0.5 – 1.2 | Male: 84.3  Female: 72.3 | Male: 50.6  Female 41.0 | Omnivorous: Insects, earthworms snails, small vertebrates (frogs, reptiles and birds), carcasses of small mammals, fruit | All measurements: K J.-C. Pei (unpublished data).  Diet: [11, 21]. |
